# Supplementary material for: Implementation of the Synergy Tool: A Potential Intervention to Relieve Health Care Worker Burnout
Source: Int J Environ Res Public Health. 2022 Dec 28;20(1):489. doi: 10.3390/ijerph20010489 (PMC9819893; doi:10.3390/ijerph20010489)
Supplement: Supplementary file 1 [file ijerph-20-00489-s001.zip › ijerph-2121100-supplementary.pdf]

Supplementary Material SI:

Table S1. Adapted Synergy Tool for Vascular

	**Acuity**					**Dependency**		
	Stability<br>*The ability to maintain a steady state.*<br>*(e.g. requiring additional monitoring)*	Complexity<br>*Severity of systems involvement*	Predictability<br>*Predictability of illness trajectory.*	Resiliency<br>*The ability to return to baseline or improve QoL.*	Vulnerability<br>*Susceptibility to stressors.*<br>*(e.g. risk of harm to self or others)*	Capacity to make decisions<br>*The ability to engage in decisions.*<br>*(e.g., **relating to pts, families, advocates**)*	Capacity to perform ADLs<br>*The ability to engage in ADLs.*<br>*(e.g. internal support)*	Resource availability<br>*The extent of resources.*<br>*(e.g. external support)*
**High need (1-2)**	• Vital signs Q2H or more frequent<br>• Sudden significant change in VS and/or CWMS and/or neuro status<br>• Deterioration in LOC<br>• RR ≤10 or ≥30<br>• HR <60 or >100<br>• SpO2 ≤88% requiring increasing oxygen needs<br>• SBP <90 or >180 mmHg<br>• Temperature ≤36.0 or ≥38.0<br>• APS monitoring<br>• Critical lab values<br>• Uncontrolled or ineffective pain control needing Q1-2H analgesia<br>• Post-op dressing requiring changing within first 24 hours<br>• Actively bleeding requiring manual pressure<br>• New minimal urine output (<20 mL/hr x 4 hrs)<br>• CIWA Q1H (score above 10)<br>• Initiating post falls protocol<br>• Blood sugars 2.0 or less	• Significant involvement of multiple systems OR complex involvement of one system requiring interventions or investigations<br>• Requiring multiple consults including Physicians, Diabetic Nurse, Allied Health members<br>• CVC in situ (PICC/femoral/jugular)<br>• Epidural/PCA/nerve block in situ<br>• 3 or more infusions<br>• Presence of 3 or more: Foley, surgical drain and/or IR drain<br>• NG or en-tube insertion<br>• Initiating anticoagulant infusion protocol<br>• Massimo monitoring<br>• Complex wounds<br>• Wound pressure relieving challenges requiring daily interdisciplinary team collaboration<br>• Wound requiring serial mechanical debridement	• Significant deviation from current course of recovery<br>• Unexpected complication requiring urgent intervention	• No coping skills<br>• Low motivation<br>• Not accepting of new condition or treatment plan	• Unmanaged mental health and addictions (MHSU) related issues<br>• Violent or aggressive behaviours (verbal or physical)<br>• Communication challenges<br>• Immunocompromised	• No capacity to make decisions with **no** Temporary Substitute Decision Maker (TSDM) available<br>• Family conflict with decision-making	• 2P assist for:<br>○ Care/ADLs<br>○ Repositioning<br>○ Mobility<br>○ Transfers<br>• Extensive and repeated coaching and education required for patient and/or family	• No or limited access to resources and supports<br>• Extensive discharge planning required<br>• Advanced equipment needs<br>• Home environment unsuitable for current needs<br>• Lack of financial support<br>• Patient needs are beyond community services and healthcare system
**Examples**	* **First 24 hours post-op**<br>* **New admission from HAU/ICU**	* **Negative pressure wound therapy (VAC or Veraflow)**	* **Post-op bleeding, hematoma, dehiscence**	* **Overwhelmed**<br>* **Fluctuating emotions**	* **Elopement risk**<br>* **Chemical or physical restraints in use**	* **Delirium**<br>* **Recent CVA**<br>* **Certified x2**	* **Ceiling lift**<br>* **CVA with deficits**	* **No fixed address**<br>* **No medical coverage**

	*** Open procedures (eg. carotids, aorta bi-fem, open AAA, TOS, TEVAR)**<br>*** Retroperitoneal bleeding**<br>*** Critical limb ischemia**<br>*** Non-rebreather or high flow oxygen**<br>*** Unexpected chest pain**<br>*** DKA**	*** Deep tunneling or unable to visualize wound base**<br>*** Multiple impairments requiring comprehensive assessment and multiple trials of interventions for off-loading (OT/PT involved)**<br>*** CAPD**<br>*** New diabetic diagnosis**<br>**• TOS drains needing Q1H priming/unpriming**<br>*** Chest tube**	*** Post-op DVTs, stroke, MI, PE**<br>*** Delirium**<br>*** Sepsis**<br>*** Post-op fall in new amputee**<br>*** Critical limb ischemia**<br>*** Compartment syndrome**	*** Non-adherence**	*** Language barrier**<br>*** Aphasia**<br>*** Renal transplant**<br>*** CIWA protocol scoring 10 or greater**		*** Recent transfer from ICU/HAU**	*** Not eligible for home health services**
**Moderate need (3)**	• Vital signs Q4H-QID<br>• Pain controlled with Q3-4H analgesia<br>• CIWA monitoring Q4H (score 9 or less)<br>• Abnormal lab values requiring intervention<br>• Blood sugar 2.1 – 4.0 or greater than 20.0	• Multiple system involvement requiring monitoring<br>• Ongoing management by multiple specialties<br>• Presence of 1-2 infusions and/or 1-2 tubes or drains<br>• Wound +/- pressure relieving with established care plan<br>• Managing urinary retention<br>• Abdominal distention<br>• Nausea and/or vomiting	• Minor deviation from current course of recovery requiring investigation<br>• Return to OR for scheduled intervention within same admission	• Reduced or unknown capacity to cope<br>• Requiring lots of encouragement<br>• Physical or cognitive status below baseline	• Managed MHSU related issues<br>• Unknown risk factors<br>• Communication strategies in place<br>• Cultural/spiritual beliefs conflict from conventional healthcare practices<br>• At risk for falls<br>• Bed rest equal to or greater than 24 hours<br>• Sleep disturbance<br>• Hallway patient	• Questionable capacity to make decisions<br>• No capacity to make decisions but has TSDM available<br>• Requires family meeting	• Supervision to 1P assist<br>• Mobility status unclear<br>• Weight-bearing status unclear<br>• Some coaching and education required for patient and/or family	• Unknown or questionable family or social supports<br>• Resource responsibility confusion (eg. WSBC, ICBC)<br>• May need equipment, home adaptations, community services and/or financial support
**Examples**	*** Electrolyte replacement**	*** Followed by ID**<br>*** Standard fluid bolus for renal protection**<br>*** Diabetic management**<br>*** Bladder scanning/TOV**	*** Surgical site infection**<br>*** Post-op ileus**<br>*** Constipation**	*** Requires various modalities to manage anxiety**<br>*** Failure to thrive**	*** Specific cultural/spiritual practices**<br>*** CIWA scoring 9 or less**<br>*** Sensory aids not available**<br>*** AVB Alert**<br>*** Bed alarm**<br>*** Poor health literacy**	*** Refusing care, rationale uncertain or unknown**<br>*** Not following weight-bearing orders**<br>*** Public Guardian and Trustee as decision maker**	*** Education needed for:**<br>**○ NPWT**<br>**○ Anticoagulant**<br>**○ Weight-bearing**<br>**○ Mobility aides**<br>**○ Drains**	*** Referred to rehab/PATH but not yet accepted**
**Low need (4-5)**	• Vital signs Q shift<br>• Pain controlled with less than Q4H analgesia	• Managed comorbidities<br>• Closed, non-draining incision	• No deviation from current course of recovery	• Effective coping skills<br>• Acceptance	• Able to communicate clearly<br>• No safety concerns<br>• Sleeping well	• Presumed capable unless otherwise demonstrated<br>• May refuse care but has a sound rationale	• Patient fully independent or minimal assist	• Has all required resources and supports in place<br>• Accepted for ALC
**Examples**		*** Dry dressing, open to air**				*** Orders to self-dose insulin**		

Supplementary Materia SI

Table S2 Adapted Synergy Tool for General Surgery Patients

	**Acuity**					**Dependency**		
	Stability<br>*The ability to maintain a steady state.*	Complexity<br>*Severity of systems involvement*<br>*(e.g. multiple issues within one or more systems)*	Predictability<br>*Predictability of illness trajectory.*	Resiliency<br>*The ability to return to baseline or improve QoL.*<br>*(e.g. internal support)*	Vulnerability<br>*Susceptibility to stressors.*<br>*(e.g. risk of harm to self or others)*	Capacity to make decisions<br>*The ability to engage in decisions.*<br>*(e.g., relating to pts, families, advocates)*	Capacity to perform ADLs<br>*The ability to engage in ADLs.*<br>*(e.g. internal support)*	Resource availability<br>*The extent of resources.*<br>*(e.g. external support)*
**High need (1-2)**	• Labile vital signs (new onset SBP < 90 mmHg, HR > 110 bpm, RR < 10 or > 20, SpO2 < 92%)<br>• Vital Signs Q15min-2H<br>• Sedation score 3-4<br>• Pain not controlled requiring Q15-30 min checks<br>• Minimal urine output (<20 mL/hr x 4 hours)<br>• Requiring blood transfusion and/or fluid resuscitation<br>• Incisions or wounds requiring continual pressure<br>• Critical lab results<br>• High flow oxygen needs	• Multiple comorbidities requiring interventions or investigations<br>• Specialists consultations<br>• CVC in situ (PICC/IVAD/femoral/jugular)<br>• Epidural in situ<br>• 3 or more infusions<br>• Presence of 3 or more NG, Foley catheter, surgical drain, or IR drain<br>• Initiating anticoagulant infusion protocol<br>• Massimo monitoring<br>• Initiating TPN or tube feeds<br>• Complex wounds<br>• Fistulas<br>• Leaking ostomy that requires change at least once a day	• Significant deviation from expected course of recovery requiring additional assessment and intervention<br>• Requiring critical care intervention	• No coping skills<br>• Lack of motivation<br>• No family coping skills<br>• Severe malnutrition<br>• Compromised immune system<br>• Acute flare-up of autoimmune disease<br>• Physical or cognitive deconditioning	• Mental Health and addictions related issues<br>• Violent or aggressive behavior (verbal or physical)<br>• Chemical or physical restraints in use<br>• Communication challenges<br>• Low health literacy<br>• Recent fall<br>• Poor sleep	• No capacity to make rational decisions without Temporary Substitute Decision Maker<br>• Deemed incompetent with no TSDM<br>• Family conflict with decision-making	• Total bed rest and repositioning required<br>• Total care for ADLs<br>• 2P+ assist for mobility<br>• Extensive and repeated coaching and education required for patient or family engaging in ADLs<br>• Full assist with ostomy care	• No or limited access to resources and supports<br>• Home environment unsuitable for current care needs<br>• Patient needs are beyond existing current community services<br>• Extensive discharge planning required
**Examples**	* **Whipple surgery**<br>* **Hepatectomy**<br>* **Pancreatectomy/splenectomy**<br>* **Colon resection with APR**<br>* **Urological surgeries**<br>* **Mastectomy drain output greater than baseline within first 24 hours**<br>* **Necrotizing pancreatitis**<br>* **Trunk traumas**	* **ICU/HAU step-down**<br>* **Deep or tunneling wound +/- moisture or drainage, +/- skin folds, and/or requiring 2+ RNs for assessment and procedure**<br>* **Hospital acquired infections (MRSA, CDI, CPO, COVID, HAUTI, PNA, surgical site infection)**<br>* **Patient going for multiple tests off unit with supervision**	* **Anastomotic leaks**<br>* **Developed PEs, DVTs, CVAs, arrhythmias**<br>* **Septic shock**	* **Overwhelmed**<br>* **Labile emotions in patient and/or family members**<br>* **Caregiver burnout**<br>* **Frequent unwarranted requests**<br>* **Undergoing immunotherapy**	* **Failure to thrive**<br>* **Language barrier**<br>* **Hard of hearing and no hearing aids present**<br>* **Visual impairment**<br>* **Post falls assessment**	* **Unconscious**<br>* **Delirium**	* **Teaching required**<br>○ **Tube feed**<br>○ **Drain**<br>○ **Ostomy**<br>* **Ceiling lift transfer**	* **No fixed address**<br>* **No medical coverage**

Supplementary Materia SI

Table S2 Adapted Synergy Tool for General Surgery Patients

**Moderate need (3)**	• Vital signs Q4H<br>• Sedation score 1-2<br>• APS monitoring<br>• Q4H pain assessment (if not APS)<br>• Hourly urine output checks<br>• Post-operative nausea/vomiting<br>• Abnormal lab results<br>• Fluid and electrolyte replacement<br>• Abnormal ECGs<br>• Post-op basic wound care	• Multiple system involvement requiring monitoring<br>• 1-2 infusions and Foley and 1 surgical drain in situ<br>• TPN at goal rate<br>• Enteral feeds at goal rate<br>• Tolerating less than 50% of baseline diet<br>• Daily dressing changes<br>• New ostomy	• Minor deviation from expected course of recovery that requires some investigation<br>• Potential for higher level of care	• Reduced or unknown capacity to cope<br>• Unknown family coping skills<br>• Moderate malnutrition<br>• Managed autoimmune disease	• Unknown risk factors<br>• Potential for violence or aggression<br>• Flight risk<br>• Communication strategies in place<br>• Limited health literacy<br>• Intermittent sleep	• Questionable capacity to make rational decisions<br>• No capacity to make decisions but has TSDM<br>• Requires family meeting	• Supervision to 1P for ADLs<br>• Some coaching and education required for patient or family engaging in ADLs<br>• Moderate assist with ostomy care	• Resource needs require further investigation<br>• Support needs require further investigation<br>• Conflicting resource responsibilities (eg. ICBC, WSBC)
**Examples**	* **High output ostomy**<br>* **Post op day 1 wound care**<br>* **Delirium**	* **Wound requiring VAC with shallow, small area, easy to seal**<br>* **VAC dressing requiring 1 RN + other for positioning**<br>* **Chest tubes**	* **Post-op ileus**<br>* **Controlled sepsis**	* **Require additional supports in order to cope**<br>* **Additional time needed to process information**	* **AVB alert**<br>* **CIWA protocol**<br>* **At risk for falls**	* **Refusing care/rationale uncertain or unknown**<br>* **Public Guardian and Trustee as decision maker**	* **1P assist getting out of bed due to tubes/lines**<br>* **Teaching in progress**	* **May need equipment, home adaptations, community services and/or financial support**<br>* **ALC planning**<br>* **Awaiting specialty treatment bed**
**Low need (4-5)**	• Vital signs stable (Q6-12H)<br>• Pain is well controlled<br>• Fluid balance and labs stable<br>• No nausea and vomiting	• 1+ systems or comorbidities that respond to interventions<br>• 0 - 1 peripheral IV<br>• Tolerating minimum 50% of oral diet<br>• Ostomy change as per care plan	• No deviation from expected course of recovery	• Ability to cope<br>• Minor or no concerns with family<br>• Minimal concerns with nutrition status	• Cooperative with care<br>• No communication challenges<br>• Adequate health literacy<br>• Sound sleep<br>• No safety concerns	• Able to make rational decisions<br>• May refuse care but has a sound rationale	• Patient/family fully independent or minimal assist with ADLs<br>• Patient/family independent or minimal assist with stoma care	• Has all required resources and supports in place<br>• Accepted to ALC facility and waiting for bed offer

Supplementary Materia SI

Table S2 Adapted Synergy Tool for General Surgery Patients

**Examples**		• **Urinary retention controlled with Flomax**					* **Standby assist**	* **Return to home/facility**

Supplementary Material SI:
Table S3. Adapted Synergy Tool for Neuro Surgery Patients

	Acuity					Dependency		
	Stability<br>*The ability to maintain a steady state.*	Complexity<br>*Severity of systems involvement*	Predictability<br>*Predictability of illness trajectory.*	Resiliency<br>*The ability to return to baseline or improve QoL. (e.g. internal support)*	Vulnerability<br>*Susceptibility to stressors. (e.g. risk of harm to self or others)*	Capacity to make decisions<br>*The ability to engage in decisions. (e.g., relating to pts, families, advocates)*	Capacity to perform ADLs<br>*The ability to engage in ADLs. (e.g. internal support)*	Resource availability<br>*The extent of resources. (e.g. external support)*
**High need (1-2)**	• **Labile** vital signs<br>• **New** fluctuations of vital signs<br>• **Significant** change in baseline LOC<br>○ GCS score < 12<br>○ Pupillary/motor response<br>• **Neuro-vitals and vitals** (Q1H or more frequent)<br>• **Actively seizing**<br>• **Difficult airway**<br>• **Increased O_2** needs<br>• **Increased airway suctioning**<br>• **Increased monitoring**<br>• **Significant neurological change**:<br>○ Sensation<br>○ Mobility<br>○ Motor strength	• **Significant/severe involvement of one or more systems**<br>• Multiple or unmanaged co-morbidities<br>• Complicated, intensive treatments/meds/ interventions requiring advanced, expert knowledge and skills<br>• **Multiple pending, active** consultations	• **Significant deviations** from expected course of recovery<br>• **Emergent, acute brain** injury or disease<br>• **Cranial vault entry**<br>• **Recent interventions**	• **Unlikely** to return to baseline<br>• **No coping** skills<br>• **No/little health literacy**<br>• **No supports**<br>• **Compromise**<br>○ malnutrition<br>○ immunity<br>○ physical or cognitive deconditioning	• **Significant Impairment**<br>○ cognition/memory<br>○ communication<br>• **Language barrier**<br>• **Mental health and/or addictions diagnoses**<br>• **Significant decreased insight**<br>• **Escalating aggressive behaviour**<br>• **High dependency** on others for care<br>• **Inability to seek support** or assistance<br>• **Sleep disturbances**<br>• **Immobility**	• **No capacity** to make rational decisions<br>• **No TSDM**<br>• **Conflicting opinions** on major care decisions	• **No capacity** to do ADLs<br>• **No support** to do ADLs<br>• **Multiple health disciplines needed** (e.g. allied health, nursing)<br>• **2 or more people for physical care**<br>• **Extensive coaching**/teaching support for ADLs is needed	• **No/limited supports**<br>• **Extensive discharge planning** required<br>• **Advanced equipment needs**<br>• **Home environment unsuitable** for current care needs<br>• **Lack of financial** support<br>• **Patient needs are beyond community services** and healthcare system (e.g. home health, rehab)<br>• **Intense family dynamics**
**Examples**	* **Subarachnoid hemorrhage**<br>* **On-call for urgent neurosurgery**<br>* **Emergency mannitol**<br>* **EVD / Lumbar drain**<br>* **Tracheostomy –** frequent suctioning and O_2 sat monitoring<br>* **Post-laminectomy hematoma**<br>* **Unstable C-Spine fractures**<br>* **Postoperative OSA**	* **Multi-trauma**<br>* **Fluid & Electrolyte Imbalances**<br>* **Dysphagia –** aspiration risk<br>* **Subarachnoid hemorrhage patient** with cardiac complications and/or fluid & electrolyte imbalances	* **TBI**<br>* **Craniotomy**<br>* **Emergency coiling**<br>* **Unstable spine fracture**<br>* **Basal skull fracture**<br>* **Brain tumor**<br>* **Delirium**<br>* **CSF leak**<br>* **Trach decannulation**<br>* **EVD removal**	* **Failure to thrive**<br>* **Frail senior**<br>* **Dementia**<br>* **Diffuse axonal injury**<br>* **Poor prognosis TBI**	* **TBI**<br>* **Elopement**<br>* **Suicide risk** or history of suicide attempts<br>* **Aphasia**<br>* **Skin breakdown**<br>* **Pneumonia**			* **Family/social supports unwilling, unable to help**<br>* **Ineligible for Canadian HC, no PHN**

Supplementary Material SI:
Table S3. Adapted Synergy Tool for Neuro Surgery Patients

	* **Anterior Cervical surgery**<br>* **Code Blue** (past 24hrs hrs)							
**Moderate Need (3)**	• **Neuro vital/vital** checks Q4H<br>• **O2** monitoring Q2-3H<br>• **Airway** suctioning Q2-3Hhr<br>• **Low GCS** (scores<12) for 24-72 hrs<br>• **Subtle neurological changes in baseline**:<br>o GCS<br>o Pupillary response<br>o Sensation<br>o Motor strengths<br>o Mobility	• **Ongoing investigation** of systems<br>• **Requires further investigation and/or monitoring** due to a system change<br>• Treatments/meds/ interventions requiring **proficient knowledge** and skills<br>• **Managed comorbidities**<br>• **Drains and tubes recently removed**<br>• **Established care plan**: post-consultations<br>• **Evidence of cognitive and/or motor improvement**	• **Subtle deviation** from expected course of recovery<br>• **Any type of post-op infection**<br>• **Planned surgery**	• **Potential** to return to baseline or **improve**<br>• **Unknown** recovery potential<br>• **Some adaptive coping** skills<br>• **Some supports**<br>• **Some health literacy**	• **Impairment- some/mild**<br>o cognition/memory<br>o communications<br>o language barrier<br>• **Some/mild elopement risk**<br>• **Decreased insight**<br>• **Some/mild aggressive behaviours**<br>• **Managed or unknown mental health** and addiction diagnoses<br>• **Some/mild dependency** on others for care<br>• **Some inability to seek support** or assistance<br>• **Some sleep disturbance**	• **Some or unknown** capacity to make **rational decisions**<br>• **-Refusing care/Rationale uncertain or unknown**<br>• **Occasional conflict** in major care decisions<br>• **Patients with legal directives**	• **Some capacity** to do **ADLs**<br>• **Some support** to do ADLs<br>• **Multiple health** disciplines needed (e.g. allied health, nursing)<br>• **1-2 people for physical care**<br>• **Some coaching**/teaching support for ADLs	• **Unknown, questionable family/social support**<br>• **Conflicting resource responsibilities** (e.g. WSBC, ICBC)<br>• **Some discharge planning** required<br>• **More information is required** to determine resource needs<br>• **May need:**<br>o Equipment<br>o home care adaptations<br>o financial support<br>o community services<br>• (e.g. home health, rehab)
**Examples**	* **Post-op day 1 for Neuro patients**<br>* **Tracheostomy**	* **Hemodialysis patients**<br>* **Weaning and/or Corked trach**	* **Spinal fracture treated**<br>* **Linear skull fracture**		* **TBI**	* **Patient with legal directive**		* **Patient requires Home Health services**
**Low need (4-5)**	• **No change** in neuro-vitals/ vitals for 48-72 hrs<br>• **Neuro-vital/vitals** checks q8 - 12 hrs	• **No or few comorbidities**<br>• Treatments/meds/ interventions requiring **basic** knowledge and skills<br>• **Minor systems** involvement	• **No deviations** from expected course of recovery	• **Expected** return to baseline<br>• **Strong adaptive** coping skills<br>• **Adequate supports**<br>• **Strong health literacy**	• **No safety concerns** or risks<br>• **Sleeping well**<br>• **Independent** capacity to care for self	• **Making rational decisions**<br>• May refuse care but has a **sound rationale**<br>• **No conflict** in major care decisions	• **Independent** ADLs or minimal<br>• **Adequate support** for ADLs<br>• **No or minimal coaching**/teaching support for ADLs	• **Capable, willing, available** family/social supports<br>• **No additional resources** needed
**Examples**			* **VP shunt revision**					

Supplementary Material SI:

Table S3. Adapted Synergy Tool for Neuro Surgery Patients

			* **Lumbar laminectomy**						

Supplementary Material SI:

Table S4. Adapted Synergy Tool for Orthopedic Surgery

	**Acuity**					**Dependency**		
	Stability<br>*The ability to maintain a steady state.*	Complexity<br>*Severity and/or number of systems involvement*	Predictability<br>*Predictability of illness trajectory.*	Resiliency<br>*Emotional coping reserves*	Vulnerability<br>*Susceptibility to stressors. (e.g. physical or emotional) (e.g. risk of harm to self or others)*	Capacity to make decisions<br>*The ability to engage in decisions. (e.g. pts, families, advocates)*	Capacity to perform ADLs<br>*The ability to engage in ADLs inc. ability to mobilize*	Resource availability<br>*The extent of resources needed for discharge. (e.g. external support)*
**High need (1-2)**	• Vital signs Q2H or more frequent<br>• Sudden significant change in VS and/or CWMS and/or patient condition<br>• Deterioration in LOC<br>• RR <10 or >30<br>• HR <60 or >100<br>• SpO2 <90% requiring increasing oxygen<br>• SBP <90 mmHg or >180mmHg<br>• Temperature <36.0 or >38.0<br>• Sedation scale 3+<br>• Pain medication Q2H or more frequent or receiving IV analgesia PRN<br>• Free flap monitoring Q2H<br>• CIWA monitoring Q1H<br>• Critical lab values<br>• Initiating post falls protocol	• Involvement of one system and/or multiple systems requiring multiple interventions<br>• Multiple active specialist consultations<br>• Multiple pre-existing comorbidities<br>• 3 or more infusions<br>• Presence of 3 or more tubes, drains or lines<br>• Initiating TPN and/or anticoagulant infusion protocol<br>• Airborne or contact plus precautions or CPO<br>• Massimo monitoring<br>• Complex wound care	• Significant deviations from expected course of recovery<br>• Unexpected complications during hospital stay	• No coping skills for patient and/or family<br>• Low motivation<br>• Withdrawn or apathetic<br>• Not accepting of new condition or treatment plan	• Suicide risk<br>• Acute and/or unmanaged mental health and substance use related issues<br>• Significant compromise:<br>○ Cognition<br>○ Mobility<br>○ Immunity<br>○ Malnutrition<br>• Frailty<br>• Violent and/or aggressive behavior (verbal or physical)<br>• Inability to communicate care needs	• No capacity to make decisions and no Temporary Substitute Decision Maker (TSDM) available<br>• Conflicting opinions on major care decisions	• Minimal to no ability to initiate or participate in ADLs including transfers<br>• Total care or 2 or more person assist	• No or limited access to resources and supports<br>• Care needs exceed community services and healthcare system<br>• Lack of financial support<br>• Ineligible for health services<br>• Intense family dynamics<br>• Advanced equipment needs coordination required<br>• Multiple discipline coordination required<br>• Extensive coaching or teaching support required
**Examples**	*** Multi trauma with multiple disciplines involved**<br>*** New admission within 24 hours from HAU/ICU**<br>*** Compartment syndrome**<br>*** RT or ICU outreach called**<br>*** Multi-modal pain control**<br>*** Stat or urgent diagnostics**	*** Multi/poly ortho trauma**<br>*** Chest tube, NG, enteral feeding tube, Foley catheter, surgical drains, central lines**<br>*** Multi-modal pain control**<br>*** Wired jaws**<br>*** HD or CAPD**	*** Fall in hospital with injury**<br>*** Hospital acquired infection**<br>*** Compartment syndrome**<br>*** CVA/DVT/PEs**<br>*** Delirium**	*** High or uncontrolled Anxiety**<br>*** Low frustration tolerance**	*** Severe aphasia**<br>*** Severe TBI**<br>*** Chemical or physical restraints in use**<br>*** Criminal injury**<br>*** Elopement risk**<br>*** Failure to thrive**<br>*** Police involvement**	*** Unconscious**<br>*** Certified for involuntary admission**	*** Total bed rest**<br>*** Spinal precautions**	*** No fixed address**<br>*** No medical coverage**<br>*** No Pharmacare coverage**<br>*** Not eligible for home health services**<br>*** Teaching required:**<br>**○ Collar or brace care**<br>**○ VAC**<br>**○ Wired jaw**

	* **Unexpected chest pain**<br>* **Active bleeding**<br>* **Active seizures**	* **Deep or tunneling wound +/- moisture or drainage +/- skin folds, and/or requiring 2+ nurses for assessment and procedure**<br>* **Degloving injury**<br>* **Delirium**<br>* **Unmanaged mental health concerns**	* **Substance withdrawal**					
**Moderate Need (3)**	• Vital signs Q4H-QID<br>• Sedation scale 2<br>• Pain controlled with Q3-4H narcotics<br>• CIWA monitoring Q4H<br>• Seizure precautions<br>• Abnormal lab values requiring intervention	• Pending investigations<br>• 1-2 infusions and/or 1-2 tubes, drains or lines requiring ongoing monitoring<br>• Non-approximated and/or draining wound<br>• Wound VAC<br>• Droplet or contact precautions	• Unknown or uncertain recovery potential<br>• Treatment plan in progress or being revised	• Reduced or questionable capacity to cope	• Unknown risk factors<br>• History of violence and/or aggression and/or substance misuse<br>• At risk behaviours with care plan established<br>• History of trauma<br>• Communication challenges	• Questionable capacity to make rational decisions requiring further investigation<br>• Questionable, unknown or undetermined TSDM	• Some ability to initiate or participate in ADLs<br>• 1 person assist, stand-by or supervision	• Unknown or questionable family or social supports<br>• Support and/or equipment needs require further planning and navigation<br>• Conflicting resource responsibilities (eg. WSBC, ICBC)<br>• Patient and/or family teaching in progress
**Examples**	* **New admission from PACU/ER**<br>* **Multi-modal pain Epidural or PCA or nerve block or spinal bolus**	* **Nerve block**<br>* **Trach**	* **Cardiac workup in progress**<br>* **GI workup in progress**<br>* **Autoimmune disease flare-up**		* **Language barrier**	* **Refusing care and rationale uncertain or unknown**	* **Falls risk**	* **May need equipment, home adaptations, community services and/or financial support**
**Low need (4-5)**	• Vital signs Q shift<br>• Sedation scale 1<br>• Pain controlled with less frequent than Q4H narcotics or no pain<br>• Medically cleared	• No other system involvement<br>• Managed comorbidities<br>• Approximated non-draining wound	• Following expected trajectory<br>• Established treatment plan	• No coping issues identified	• No safety concerns or risk	• Presumed capable unless otherwise demonstrated<br>• Capable of making own decisions<br>• Established TSDM	• Independent	• Has all required resources and supports in place<br>• Accepted to ALC facility and waiting for bed offer<br>• Capable, willing, available family/social supports<br>• Teaching completed and able to demonstrate back
**Examples**	* **ALC**<br>* **Admit day of surgery**	* **Admit day of surgery**<br>* **Saline lock or no IV required**						* **Ready for home**<br>* **Hospice, Rehab, CV, Path, LTC**<br>* **Logged to PTN**

Supplementary Material SII:

Example Case Vignettes from General Surgery

1. 74 years old male with a history of colon cancer with liver metasis, hypertension, GERD, arthritis, right hemicolectomy. He had laparoscopic converted to open right hepatotectomy 2 days ago. His epidural was discontinued yesterday and his current pain level is 3/10 to abdomen upon activity. He takes Tylenol regularly and required 2 times PRN analgesics for pain overnight.

2. 22 years old male admitted for lap subtotal colectomy with a history of ulcerative colitis, generalized anxiety disorder, depression, OCD, insomnia, concussion, alcohol use disorder. Patient on CIWA protocol and symptomatic withdrawal post-surgery. CIWA score between 12-15 requiring Q1H vitals and CIWA assessment. Patient confused, diaphoretic, nauseous and moderately anxious and agitated. Patient not cooperative with care at times. Patient also complains of pain to abdomen and requiring frequent PRN analgesics.

3. 61 years old female admitted for gastroparesis and diabetic ketoacidosis with a history of hypertension, cerebral aneurysm, CAD with CABG, Hep C+, diabetes, and CKD. Patient had GJ tube insertion a week ago. Culture of the insertion site sent to lab and antibiotics started for a possible infection at the GJ insertion site a week after. Patient has a PICC that is capped and used for IV antibiotics intermittently. Patient is NPO and has TF iso 1.2 admin via GJ tube continuously with water flushes. Complicated discharge planning requiring community placement and GJ tube home teaching.

4. 54 years old male. He was admitted to the hospital for Crohn's ileocolic anastomotic stricture with resection his past medical history consists of Crohn's and multiple surgeries due to complications of his Crohn's. He required an ileostomy with future plan to reanastomosis. His hospital stay is complicated by requiring IR drains for abscesses on left and right side of the abdomen. The drains are flushed with 10cc NS bid and have had high output. Patient is also receiving IV antibiotics and TPN through a PICC line due to his high output ileostomy and severe weight loss.
